# T‐type calcium channels as therapeutic targets in essential tremor and Parkinson's disease

**DOI:** 10.1002/acn3.51735

**Published:** 2023-02-04

**Authors:** Lillian G. Matthews, Corey B. Puryear, Susana S. Correia, Sharan Srinivasan, Gabriel M. Belfort, Ming‐Kai Pan, Sheng‐Han Kuo

**Affiliations:** ^1^ Praxis Precision Medicines Boston Massachusetts 02110 USA; ^2^ Department of Neurology University of Michigan Ann Arbor Michigan 48109 USA; ^3^ Department and Graduate Institute of Pharmacology National Taiwan University College of Medicine Taipei 10051 Taiwan; ^4^ Neurobiology and Cognitive Science Center National Taiwan University Taipei 10617 Taiwan; ^5^ Department of Medical Research National Taiwan University Hospital Taipei 10002 Taiwan; ^6^ Cerebellar Research Center National Taiwan University Hospital Yun‐Lin Branch Yun‐Lin 64041 Taiwan; ^7^ Department of Neurology Columbia University New York New York 10032 USA; ^8^ Initiative for Columbia Ataxia and Tremor Columbia University New York New York 10032 USA

## Abstract

Neuronal action potential firing patterns are key components of healthy brain function. Importantly, restoring dysregulated neuronal firing patterns has the potential to be a promising strategy in the development of novel therapeutics for disorders of the central nervous system. Here, we review the pathophysiology of essential tremor and Parkinson's disease, the two most common movement disorders, with a focus on mechanisms underlying the genesis of abnormal firing patterns in the implicated neural circuits. Aberrant burst firing of neurons in the cerebello‐thalamo‐cortical and basal ganglia‐thalamo‐cortical circuits contribute to the clinical symptoms of essential tremor and Parkinson's disease, respectively, and T‐type calcium channels play a key role in regulating this activity in both the disorders. Accordingly, modulating T‐type calcium channel activity has received attention as a potentially promising therapeutic approach to normalize abnormal burst firing in these diseases. In this review, we explore the evidence supporting the theory that T‐type calcium channel blockers can ameliorate the pathophysiologic mechanisms underlying essential tremor and Parkinson's disease, furthering the case for clinical investigation of these compounds. We conclude with key considerations for future investigational efforts, providing a critical framework for the development of much needed agents capable of targeting the dysfunctional circuitry underlying movement disorders such as essential tremor, Parkinson's disease, and beyond.

## Introduction

Essential tremor (ET) and Parkinson's disease (PD) are the two most common movement disorders worldwide. In the United States, an estimated 7 million individuals have ET, representing approximately 2% of the total population,^1^ which is slightly higher than reported global prevalence estimates of 0.32–1.33%.^2^, ^3^ Prevalence of ET increases with age, with rates reaching 5.8% in patients aged ≥65 years and estimated to increase ~1.7‐fold with every decade of life; indeed, rates exceeding 20% have been observed in the 9th and 10th decades of life.^2^ Prevalence estimates for PD indicate there are approximately 1 million affected individuals aged ≥45 years in the United States, with prevalence increasing from 0.4% in patients aged 65–74 years, to nearly 2% in those >80 years.^4^, ^5^ Importantly, with an increasingly aging population, novel approaches for treating neurologic diseases are paramount. A large focus of ET and PD research has centered around the degeneration of specific cell populations,^6^, ^7^, ^8^, ^9^, ^10^ with the objective of developing disease‐modifying therapies, although, to date, no such interventions have been approved.^11^, ^12^ More recently, there has been increasing interest in targeting dysregulated neuronal activity as a novel and promising approach to treating ET and PD.

Aberrant neuronal activity has been implicated in the pathology of several neurologic conditions, including epilepsy, tinnitus, neuropathic pain, dystonia, ET, PD, and major depression.^13^, ^14^, ^15^, ^16^, ^17^ The role of dysfunctional circuitry is particularly well studied in ET and PD.^18^, ^19^, ^20^ Specifically, the cerebello‐thalamo‐cortical (CTC) and basal ganglia‐thalamo‐cortical (BG) circuits play pivotal roles in motor function, with related CTC and BG disturbances widely implicated in ET and PD, respectively.^21^, ^22^, ^23^, ^24^, ^25^, ^26^ Abnormal burst firing and dysregulated oscillatory neural activity have been identified as important factors mediating tremor and motor symptoms in both conditions.^18^, ^27^, ^28^, ^29^, ^30^, ^31^, ^32^ Surgical ablation and deep brain stimulation (DBS) provide supportive evidence for firing pattern modulation in ET and PD. Targeted disruption of the thalamic ventral intermediate nucleus (VIM) via DBS can reduce burst firing in patients with ET and improve tremor symptoms.^33^, ^34^, ^35^ Additionally, surgical thalamic ablation can improve symptoms in both ET and tremor‐dominant PD.^36^, ^37^ In patients with PD, DBS of the internal globus pallidus (GPi) or subthalamic nucleus (STN) can improve rigidity, whereas bradykinesia can be improved by targeting the STN.^38^ Despite success in many patients, DBS is not without risks common to surgical interventions, including perioperative hemorrhage and infection, with reduced efficacy also observed following long‐term use.^39^, ^40^, ^41^, ^42^ Consequently, treatment options become limited as the disease advances,^43^ implicating an urgent need for targeted, noninvasive pharmacologic approaches that can modulate the aberrant circuit activity in ET and PD and provide symptomatic relief.

The search for pharmacologic options has led to identification of various approaches to modulating neural circuit activity in the CTC and BG. One of the most promising targets are T‐type calcium channels (TTCC), known to be involved in the generation of burst activity in several brain areas.^44^, ^45^, ^46^ Notably, TTCCs are highly expressed in specific structures of CTC and BG circuits and play an important role in modulating neuronal action potential firing patterns.^47^, ^48^, ^49^, ^50^, ^51^, ^52^, ^53^ Furthermore, increasing evidence highlights their role in the aberrant bursting phenomenon observed in ET and PD,^18^, ^29^, ^54^ with TTCC blockade shown to be capable of inhibiting this bursting phenotype and ameliorating symptoms in animal models of both ET and PD.^29^, ^54^, ^55^, ^56^ Here, we discuss in depth the mechanistic role of TTCCs in the pathophysiology of ET and PD. More specifically, we review the evidence supporting the potential for TTCC blockade as a pharmacologic approach to symptomatic therapy, with the goal of informing future efforts in developing TTCC‐targeted therapies for patients with ET and PD.

## TTCC Overview and CNS Expression Patterns

TTCCs—named “T‐type” due to their transient calcium conductance—are low voltage‐activated calcium channels with key roles in membrane excitability.^57^, ^58^ These channels allow calcium entry into cells at relatively hyperpolarized membrane potentials (Ca_V_3; −70 to −55 mV)^58^, ^59^ compared with high voltage‐activated calcium channels, which activate and allow calcium entry at more depolarized membrane potentials (Ca_V_1 and Ca_V_2; > −40 mV).^58^ A brief hyperpolarizing event within a single cell is sufficient to activate TTCCs, facilitating depolarization of the membrane and activation of low‐threshold calcium current leading to generation of low voltage‐activated calcium spikes, otherwise known as rebound bursting.^59^ This voltage sensitivity allows TTCCs to support a window current that is near the resting membrane potential, in turn regulating neuronal excitability.^60^, ^61^

**Figure 1:**
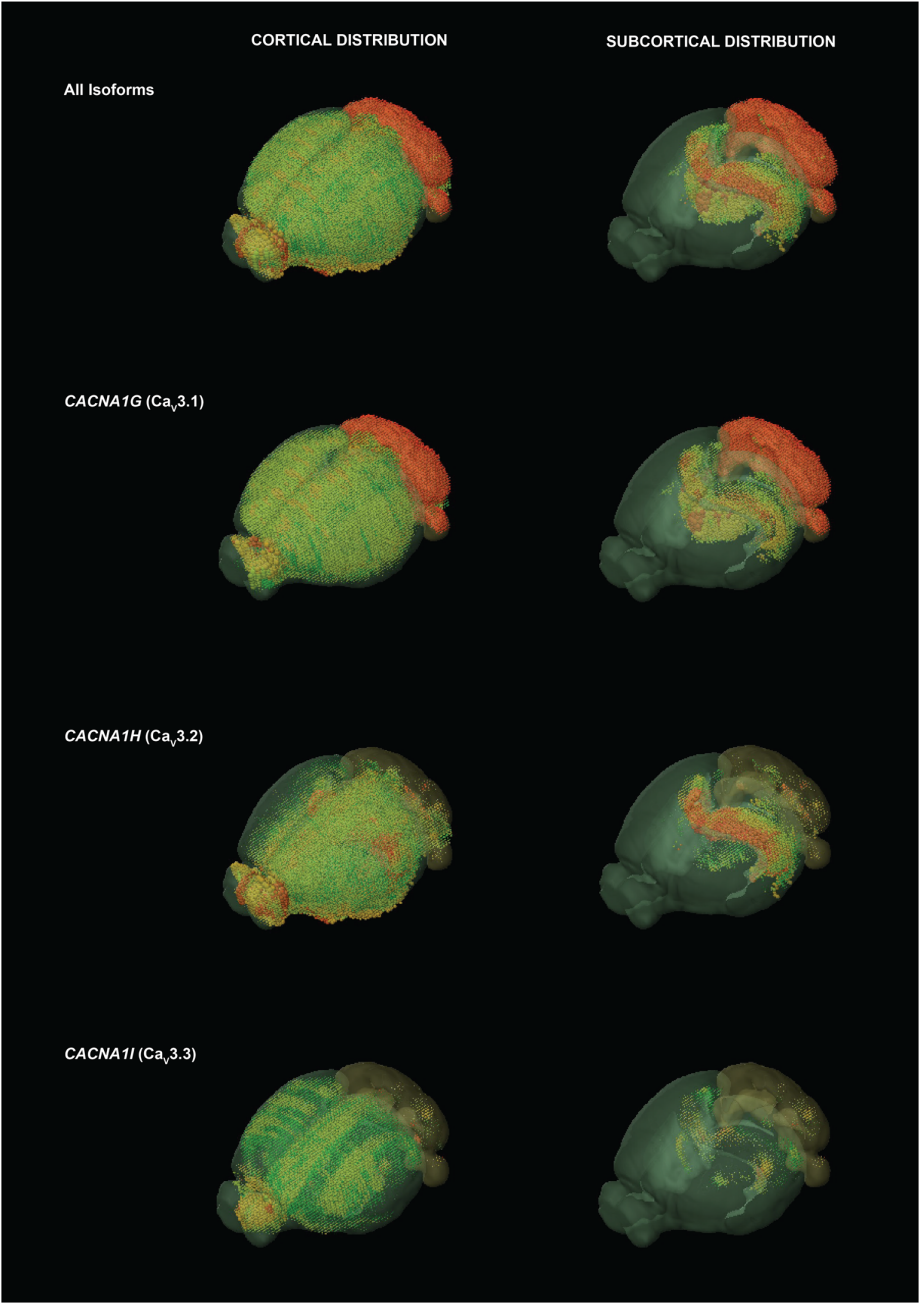
Gene expression patterns of TTCCs in mouse brain. Three‐dimensional gene expression patterns of *CACNA1G* (Ca_V_3.1), *CACNA1H* (Ca_V_3.2), and *CACNA1I* (Ca_V_3.3) in mouse whole brain. Gene expression patterns for each isoform are shown for all brain regions on the *left* (cortical distribution visible), and for regions of greatest subcortical expression on the *right*—cerebellum, thalamus, and hippocampus. Red and green denote regions of highest and lowest gene expression levels, respectively. The greatest expression of Ca_V_3.1 is in the cerebellum and the greatest expression of Ca_V_3.2 is in the hippocampus. *Images created from the Allen Mouse Brain Atlas using Brain Explorer 2.0*.

**Figure 2:**
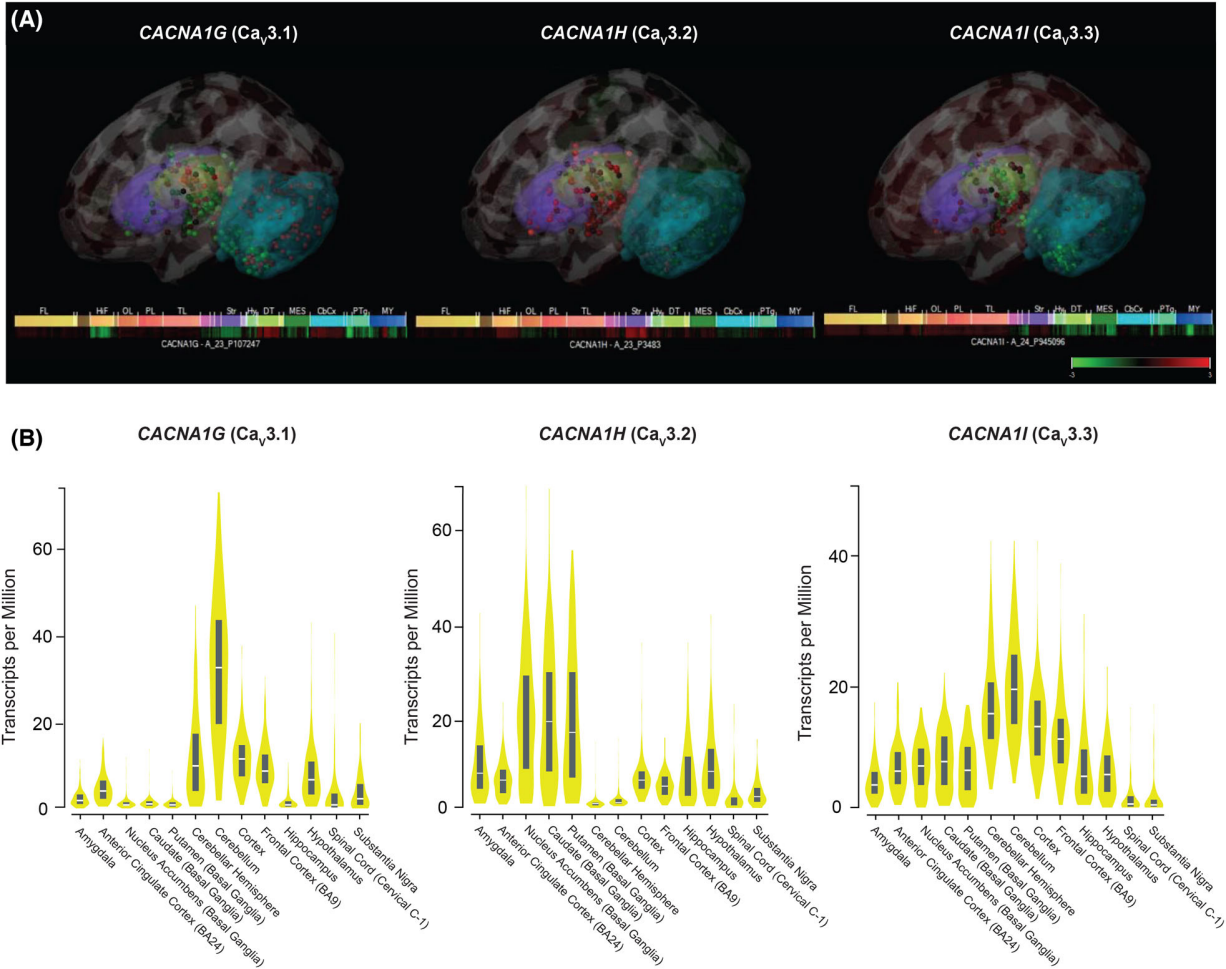
Gene expression patterns of TTCCs in human brain. (A) Microarray‐based gene expression of *CACNA1G* (Ca_V_3.1), *CACNA1H* (Ca_V_3.2), and *CACNA1I* (Ca_V_3.3) in human brain. Three‐dimensional expression patterns from a representative donor brain are shown for each isoform, overlaid on basal ganglia (purple), thalamus (green), and cerebellum (cyan). Data are normalized across the entire set of microarray samples and shown as z‐scores. *Images created from the Allen Human Brain Atlas using Brain Explorer 2.0*. (B) Violin plots depicting in situ hybridization‐based gene expression of *CACNA1G* (Ca_V_3.1), *CACNA1H* (Ca_V_3.2), and *CACNA1I* (Ca_V_3.3) in human brain. Gene expression values are shown in transcripts per million, calculated from a gene model with isoforms collapsed to a single gene. No other normalization steps have been applied. Box plots are shown as median and 25th and 75th percentiles. *Plots generated and adapted from The Genotype‐Tissue Expression (GTEx) Project portal*.

Ca_V_3.1,^62^ Ca_V_3.2,^63^ and Ca_V_3.3^64^ constitute the three known TTCC isoforms, each with unique biophysical features and distribution patterns.^62^, ^63^, ^65^, ^66^, ^67^, ^68^ In humans, Ca_V_3.1, Ca_V_3.2, and Ca_V_3.3 are encoded by the genes *CACNA1G* (chromosome 17), *CACNA1H* (chromosome 16), and *CACNA1I* (chromosome 22), respectively.^69^ TTCCs are expressed widely throughout the brain and in peripheral tissues, with particularly high expression within cerebellar motor circuits and tremor‐related structures (Figures 1 and 2, Figure S1).^66^, ^70^, ^71^, ^72^, ^73^, ^74^, ^75^ Ca_V_3.1 channels are expressed in the soma and dendrites of neurons in several brain regions including the cerebellum, thalamus, hypothalamus, olfactory bulb, basal ganglia, cortex, and hippocampus, as shown by rodent and human data.^62^, ^65^, ^66^, ^67^, ^71^, ^72^, ^73^, ^74^, ^75^ One of the key functions of Ca_V_3.1 is to regulate the oscillatory activity of thalamic neurons, as first described in cat dorsal lateral geniculate nucleus.^76^ While neural oscillation can refer to rhythmic electrical activity of clusters of activated neurons, individual neurons demonstrate rhythmic oscillation, as shown in the work of McCormick and Pape via intracellular recordings within individual cat dorsal lateral geniculate relay neurons.^76^ Notable high expression levels of Ca_V_3.1 in mouse and human cerebellum^71^, ^72^, ^73^, ^74^, ^75^ suggest its potential further role in cerebellar circuit function. Although more widely expressed than Ca_V_3.1,^77^ Ca_V_3.2 channels in mouse are predominantly expressed in the hippocampus (dentate gyrus),^72^, ^73^, ^78^ while in human brain, expression appears greatest in the thalamus and basal ganglia.^71^, ^74^, ^75^ Ca_V_3.2 channels have been shown to control NMDA receptor‐mediated transmission at central synapses in cultured rat hippocampal cells expressing the human variant.^79^ Ca_V_3.2 are also required for acute pain perception^80^ and excitability of spinal cord lamina II neurons, as demonstrated in mice.^81^ Ca_V_3.3 channels are similarly expressed in various brain regions in both mouse^72^, ^73^ and human brain,^74^, ^75^ and have been shown to facilitate neuronal oscillation in mice, particularly in the thalamus, where they are key for generating sleep spindles during non‐rapid eye movement (NREM) sleep.^82^ Published data to date suggest that Ca_V_3.3, and to a lesser extent Ca_V_3.2, plays the dominant role in sleep spindle rhythmogenesis, whereas Ca_V_3.1 appears to play a minimal role in sleep spindle activity.^82^, ^83^, ^84^, ^85^ It should be noted, however, that these reports rely on indirect measurement of sleep spindles (EEG power in the sigma frequency range, 10–15 Hz). As such, a deeper analysis of specific sleep spindle features (e.g., spindle amplitude, frequency, duration, etc.) may highlight more nuanced roles each Ca_V_ isoform plays in the generation and/or regulation of sleep spindles.

**Figure 3:**
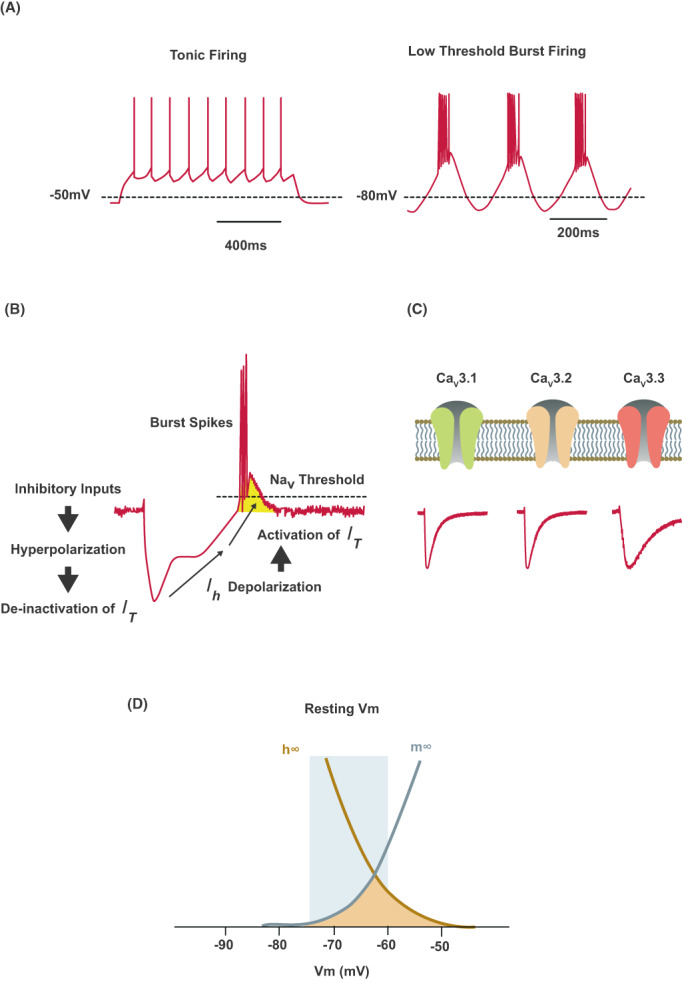
TTCC neuronal firing patterns. (A) Illustrations representing the firing patterns that are dependent on the contribution of TTCC within various brain areas and networks, including populations of thalamic, cerebellar, and cortical neurons. Tonic firing (*left*) is more likely to occur in cells with either low TTCC expression or in neurons with deactivated TTCC due to a depolarized membrane potential. Low threshold burst firing (*right*) is likely to occur in cells that have a higher density of TTCC channels, such as those in the VIM or STN, or in neurons with hyperpolarized membranes that activate TTCC. (B) Membrane hyperpolarization activates TTCC, leading to production of low threshold spikes and subsequent rebound burst firing. *Adapted from Park et al*.^86^ *as licensed under CC BY 4.0*. (C) Ca_V_3.1, Ca_V_3.2, and Ca_V_3.3 current traces elicited by action potentials in cultured HEK‐293 cells. (D) Illustration of the window current (orange shading) of TTCCs which arises due to overlap of steady‐state inactivation and activation properties. The window current of TTCCs occurs within the range of the resting membrane potential (blue shading) and has been shown to be enlarged in patients with *CACNA1G* mutations and inhibited by TTCC blockade.^87^ (C and D) *Adapted from Lory et al*.^88^ *as licensed under CC BY 4.0*. *I*_h_, HCN channel‐mediated currents; *I*_T_, T‐type calcium current; Na_V_, voltage‐gated sodium channel; Vm, membrane potential.

A key function of TTCCs is to regulate neuronal firing patterns (Figure 3).^86^, ^87^, ^88^, ^89^ Burst firing and associated subthreshold membrane potential oscillations, mediated by TTCC activation, occur in various brain areas and networks, including populations of thalamic, cerebellar, and cortical neurons, and modulate *thalamocortical* oscillations, spike–wave discharges, and neuropathic pain.^16^, ^44^, ^90^, ^91^, ^92^, ^93^, ^94^, ^95^, ^96^, ^97^, ^98^ Of note, TTCC genetic mutations have been identified in three families diagnosed with ET and have been implicated in spinocerebellar ataxia 42, epilepsy, autism, schizophrenia, developmental disorders, and other neurologic conditions (Table 1).^87^, ^99^, ^100^, ^101^, ^102^, ^103^, ^104^, ^105^, ^106^, ^107^, ^108^, ^109^, ^110^ Given their key roles in regulating neuronal firing, pharmacologically targeting TTCCs thus has the potential to yield a wealth of therapeutic benefits in various neurologic disorders. Importantly, while various Food and Drug Administration (FDA)‐approved agents, including anticonvulsants, calcium channel blockers, and psychotropics, exhibit some degree of TTCC blockade (Table 2),^111^, ^112^, ^113^, ^114^, ^115^, ^116^, ^117^, ^118^, ^119^, ^120^ no currently approved agent selectively targets TTCCs as a primary mechanism of action for its indicated use.

**Table 1:** TTCC mutations in neurologic disorders.

Isoform	Disease	Protein‐level mutation(s)	Effect on TTCC electrophysiological properties
Ca_V_3.1/*CACNA1G* ^99^	Essential tremor	Gly627Arg Arh456Glu Arg1235Gln	Little to no difference versus wild type
Ca_V_3.1/*CACNA1G* ^100^	Spinocerebellar ataxia 42	Ala961Thr	Unknown
Ca_V_3.1/*CACNA1G* ^101^, ^102^, ^103^	Spinocerebellar ataxia 42	Arg1715His	Shift toward more positive membrane potentials (inactivation and activation)
Ca_V_3.1/*CACNA1G* ^104^	Spinocerebellar ataxia 42	Met1574Lys	Unknown
Ca_V_3.1/*CACNA1G* ^105^	Autosomal‐dominant cerebellar syndrome	Arg1715His	Shift toward more positive membrane potentials (inactivation and activation)
Ca_V_3.1/*CACNA1G* ^106^	Infantile‐onset syndromic cerebellar atrophy	Ala961Thr Met1531Val	Gain of function Gain of function
Ca_V_3.1/*CACNA1G* ^87^	Childhood‐onset cerebellar atrophy	Ala961Thr Met1531Val	Gain of function Gain of function
Ca_V_3.2/*CACNA1H* ^107^	Autism	Arg212Cys Arg902Trp GLy2886Cys Ala1874Val/Arg1871Gln	Loss of function Loss of function Loss of function Loss of function
Ca_V_3.3/*CACNA1I* ^108^	Schizophrenia	Thr797Met Arg1311His	Unknown Unknown
Ca_V_3.3/*CACNA1I* ^109^, ^110^	Schizophrenia	Thr797Met Arg1346His	Unknown

**Table 2:** FDA‐approved agents with TTCC blocking properties.

	Approved clinical uses
*Anticonvulsants*	
Ethosuximide^111^	Absence seizures
Zonisamide^112^	Partial seizures, absence seizures, infantile spasms, essential tremor (second line)
Trimethadione^113^	Absence seizures
Valproic acid^114^	Partial seizures, absence seizures, bipolar disorder, migraine
*Calcium channel blockers*	
Verapamil^115^	Hypertension, angina
Amlodipine^116^	Hypertension, coronary artery disease, angina
Nicardipine^116^	Hypertension, angina
Nimodipine^116^	Subarachnoid hemorrhage
*Psychotropics*	
Pimozide^117^	Tourette's syndrome
Fluoxetine^118^, ^119^	Major depressive disorder, anxiety disorders, obsessive compulsive disorder, eating disorders, premenstrual dysphoric disorder
Trazodone^120^	Major depressive disorder, insomnia

## TTCCs in Essential Tremor

**Figure 4:**
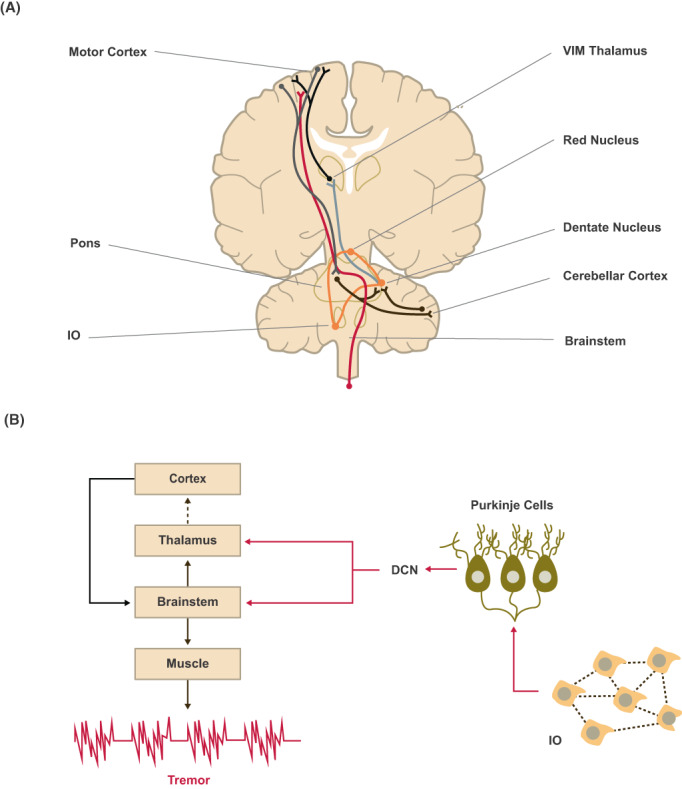
CTC circuit dysfunction in ET. (A) Key nodes of the CTC circuit (cerebellar cortex, DCN including the dentate nucleus, thalamic VIM, and motor cortex) and the intersecting white matter pathways that connect them. Orange = Guillain Mollaret triangle connecting red nucleus, IO, and dentate nucleus; red = corticospinal tract, blue = dentatothalamic tract, gray = corticopontine tract, black (infratentorial) = pontocerebellar tract, and black (supratentorial) = thalamocortical tract. (B) Tremor generation is mediated by TTCCs in IO neurons which project climbing fibers to PC dendrites within cerebellar cortex, and form synapses on glutamatergic neurons of the DCN, leading to inhibitor responses followed by rebound excitation. TTCCs play a key role in generating subsequent burst firing in the thalamus, with thalamocortical (dashed line) pathways transmitting tremor‐related signals to brainstem and spinal cord via corticopontine and corticospinal tracts. DCN, deep cerebellar nuclei; IO, inferior olive; VIM, ventral intermediate nucleus.

Essential tremor (ET) is characterized by a 4–12 Hz postural and action tremor, primarily affecting the upper limbs but often accompanied by head and voice tremors.^121^, ^122^, ^123^ Management of tremor in affected patients is complicated by multiple comorbidities and high treatment discontinuations, all of which contribute to high disease burden.^124^ Several genetic studies of families with ET suggest that an autosomal‐dominant inheritance pattern is common.^125^, ^126^, ^127^, ^128^ Variants in the *CACNA1G* gene encoding the Ca_V_3.1 isoform have been reported in three families based on whole exome (of 37 families) and whole genome (of eight families) sequencing studies of familial ET; however, corresponding functional studies showed little to no change from wild type.^99^, ^129^ Clinical and preclinical data suggest the dysregulation of neuronal activity in the CTC network in ET.^18^ Within the CTC, TTCCs are highly expressed in deep cerebellar nuclei (DCN) and Purkinje cells (PC),^6^, ^130^, ^131^ ventral thalamic nuclei (particularly the VIM),^132^ and motor cortex (Figure 4).^66^ Neuronal burst firing has been recorded in the VIM in ET patients undergoing DBS, and the firing pattern correlates with tremor.^18^ Furthermore, bilateral or unilateral VIM‐DBS has both been shown to improve tremor in patients with ET.^33^, ^34^, ^35^ The inferior olive (IO) is a related TTCC‐rich^66^ component that modulates CTC activity and may also be implicated in tremor generation.^133^, ^134^, ^135^, ^136^ While there are suggestions that broader olivo‐cerebello‐thalamo‐cortical network dysfunction exists in ET,^70^ the traditional olivary model focusing on a central IO disturbance in ET has been increasingly in question, with limited imaging or postmortem evidence to support its primary role in tremor.^137^ On the other hand, the CTC has been at the center of ET pathophysiology, and neurons at each node (cerebellar cortex, DCN, VIM, and motor cortex) are enriched for TTCC expression to modulate the firing patterns in normal and diseased states.

Animal models have provided insights into the mechanisms underlying tremor generation, the most common of which is the harmaline rodent tremor model.^138^ Harmaline, a central nervous system (CNS) stimulant, induces acute tremor in a variety of animals,^139^, ^140^, ^141^ with peak tremor frequency varying by species and ranging from 8–10 Hz in monkeys to 10–16 Hz in pigs.^141^, ^142^, ^143^, ^144^ Importantly, harmaline induces tremor by augmenting TTCC function.^145^ Both knockout and selective knockdown of *CACNA1G* have been shown to eliminate tremor in a harmaline mouse model,^146^ and TTCC blockers have shown efficacy in harmaline‐induced tremor in mice and rats in a dose‐dependent manner.^55^, ^56^, ^147^ Importantly, harmaline studies have contributed critical insights into the role of TTCC‐rich CTC nodes in tremor genesis. Subthreshold oscillations (i.e., neuronal oscillations that occur as a result of intrinsic membrane potential fluctuations but which are below the voltage threshold required to cause a neuron to fire) are dependent on TTCC conductance^148^ and closely associated with generation of tremor rhythm, and have been shown to be amplified in IO neurons following harmaline treatment.^146^, ^149^, ^150^ In harmaline‐induced tremor, hyperactive glutamatergic IO neurons recruit medial regions of cerebellar cortex via climbing fibers to PC dendrites, inducing PC burst firing rhythmically and synchronously.^151^, ^152^ Additionally, harmaline administration results in the expression of c‐fos, a marker for neuronal activation, in PC nuclei in rats, further supporting PC hyperexcitability.^153^ Climbing fiber and PC interactions appear to be particularly important in harmaline‐induced tremor. Indeed, *pcd* mice with some PC degeneration but intact climbing fibers have an attenuated response to harmaline administration, with reduced tremor frequency and amplitude relative to wild‐type mice.^154^ In contrast, *Lurcher* mice which lack PC neurons and climbing fibers do not develop tremor upon harmaline administration.^154^ Interestingly, repeated administration of harmaline to rats results in an attenuated tremor response, which may be due to a reduction in PC neuron burst firing and/or degeneration of PC neurons themselves.^155^, ^156^, ^157^ Within the DCN, dysfunction of parvalbumin‐expressing (PV^+^) excitatory neurons results in action tremor in mice, and DCN synaptic transmission block reverses the tremor.^158^ Burst firing of DCN neurons travels to the cerebral cortex via the VIM of the thalamus,^46^ with tremor manifesting as a result of rhythmic modulation of primary motor cortex signals that are conveyed to muscles. In line with this, both VIM neuronal bursting^159^, ^160^ and enhanced rhythmic neuronal activity in the primary motor cortex measured with magnetoencephalography have demonstrated coherence with tremor.^21^

The *Grid2* ^*dupE3*^ mouse model is a recently developed tremor model for ET, in which mice have a genetic mutation of the *Grid2* gene (exon 3 duplication) that leads to increased protein degradation and reduced GluRδ2 cerebellar expression levels, resembling the GluRδ2 deficiency observed in patients with ET.^22^ GluRδ2 is a synaptic organizer for PC, and the reduction of GluRδ2 levels in *Grid2* ^*dupE3*^ mice results in aberrant climbing fibers extending into parallel fiber synaptic territory,^22^ mimicking observations in the human postmortem ET cerebellum.^161^ *Grid2* ^*dupE3*^ mice develop 20 Hz cerebellar oscillatory activity (coherent with action tremor), which can be dampened by ethanol and first‐line agents used to treat patients with ET, including propranolol and primidone.^22^ Notably, IO neurons have different firing patterns, including simple spikes and bursting activity that heavily depends on TTCC, and the burst firing of IO neurons has been shown to be phase‐coupled with tremor in *Grid2* ^*dupE3*^ mice,^22^ supporting that TTCC in IO neurons may contribute to tremor. Interestingly, this mouse model may have some translational value since cerebellar oscillatory activity measured by electroencephalography (EEG) has also been described in patients with ET,^22^ with evidence suggesting that cerebellar oscillations may be a relevant biomarker of dysregulated cerebellar synaptic organization in some patient subpopulations.^162^

Despite the growing body of preclinical evidence, there has been a paucity of studies on the effects of TTCC blockers on human tremor. Moreover, few selective TTCC blockers exist. For example, 1‐octanol and its primary metabolite octanoic acid have shown evidence of tremor reduction in adults with ET.^163^, ^164^ Though 1‐octanol has been shown to block TTCC currents in vitro,^165^ as a long‐chain alcohol, the biologic mechanism of action is likely complex and similar to ethanol. The antiepileptic drug, zonisamide, was an early agent considered for ET due to its TTCC blocking properties;^166^ however, it has also been shown to modulate voltage‐gated sodium channels and GABA receptors,^167^, ^168^ and its clinical efficacy has not been consistently demonstrated.^169^, ^170^, ^171^, ^172^ Ethosuximide is another antiepileptic agent with nonselective TTCC blocking properties^173^ that has previously shown promise in mouse models of ET,^56^ but not in patients.^174^ The clinical benefit of these compounds thus remains unclear. In recent years, renewed interest in the development of more selective TTCC blockers as a therapeutic approach in ET has led to various clinical trials investigating the safety and efficacy of this mechanism.^55^, ^175^, ^176^, ^177^, ^178^ Suvecaltamide (JZP385/CX‐8998) has demonstrated some improvement in tremor and activities of daily living in a Phase 2 proof‐of‐concept study in ET patients, although the primary efficacy endpoint was not met.^175^, ^176^ Suvecaltamide is currently under evaluation in Phase 2b trials (NCT05122650). Recent reports from a Phase 2 clinical trial of NBI‐827104 indicate lack of tremor severity reduction in adult participants with ET, although it will be important to understand the full pharmacokinetic, efficacy, safety, and tolerability profile of this compound and its impact on activities of daily living. Notably, none of these trials included biomarkers of TTCC blockade; as such, it is difficult to determine whether functional TTCC blockade was achieved. PRAX‐944 is a selective TTCC blocker previously shown on cryo‐electron microscopy to have high binding affinity to the Ca_V_3.1 pore domain.^179^ A recent translational study of PRAX‐944 included measurement of sleep spindle‐related activity (sigma band EEG power) during NREM sleep as a biomarker of functional TTCC blockade, and demonstrated robust sigma band reduction (relative to other spectral frequencies) in both rodents and healthy human participants.^55^ Furthermore, recent PRAX‐944 Phase 2 results showed statistically significant and clinically meaningful improvements in both tremor severity and activities of daily living in patients with ET.^177^ PRAX‐944 is currently under evaluation in a larger Phase 2b trial.^178^ These data with more selective TTCC blockers are promising and highlight the importance of biomarkers of TTCC activity in informing selection of doses expected to produce a functional response in relevant circuitry implicated in ET.

## TTCCs in Parkinson's Disease

**Figure 5:**
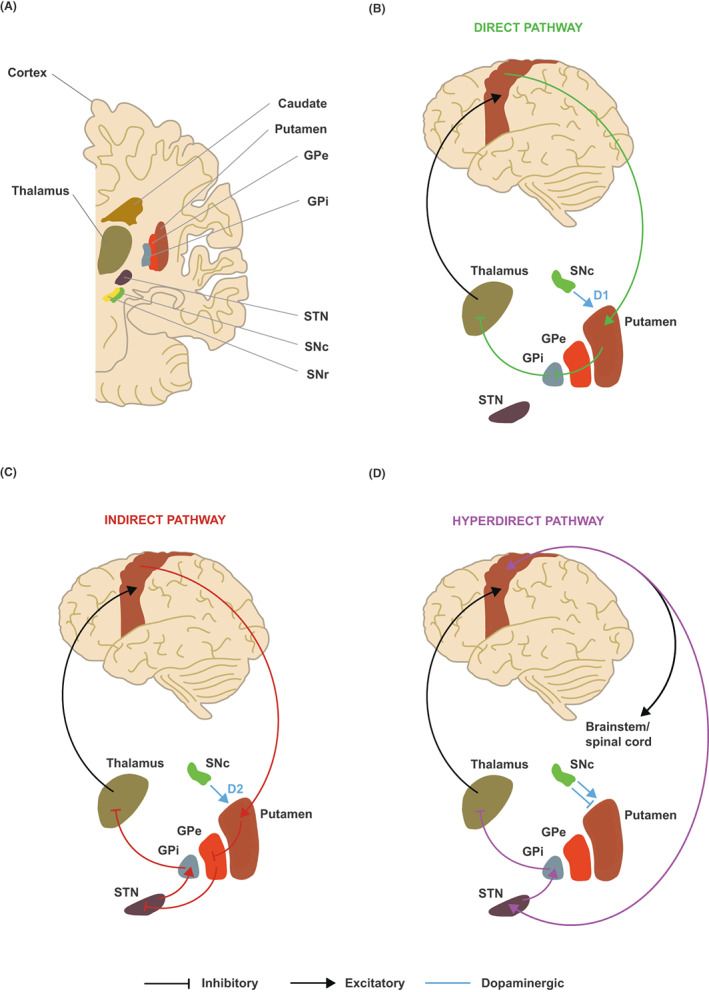
BG motor circuit dysfunction in PD. (A) Cross‐sectional illustration of BG motor circuit components. (B) In the BG motor circuit, sensorimotor cortex neurons project to the putamen. Dopamine (blue) from the SNc activates the direct (green) pathway which promotes movement in the healthy state via excitatory D1 receptors. The direct pathway projects from the putamen to the GPi, continuing to the thalamus before terminating in motor cortex via thalamocortical projections. (C) Dopamine (blue) also activates the indirect (red) pathway which suppresses movement in the healthy state via inhibitory D2 receptors. The indirect pathway projects from the putamen to the GPe, continuing to the STN, GPi, and thalamus before terminating in motor cortex. In the parkinsonian state, the loss of dopamine from the SNc leads to *hypoactivity* of the direct pathway and *hyperactivity* of the indirect pathway, with the imbalance resulting in PD hypokinetic symptoms. (D) The hyperdirect (purple) pathway projects directly from the cortex to the STN. GPe, external globus pallidus; GPi, internal globus pallidus; SNc, substantia nigra compacta; SNr, substantia nigra reticulata; STN, subthalamic nucleus. *Adapted from Hashemiyoon, et al*.^185^ *as licensed under CC BY 4.0*.

PD is clinically manifested by cardinal symptoms of bradykinesia (slowness of movement), rigidity, or stiffness that impedes normal motor activities, and/or rest tremor.^180^ The motor symptoms of PD are frequently disabled and significantly affect daily function.^181^ Although the primary pathologic mechanisms driving the neurodegenerative processes in PD have not yet been fully identified, the hypokinetic symptoms, bradykinesia, and rigidity have been linked to the loss of dopamine (DA) neurons within the substantia nigra pars compacta (SNc) and the resulting reduction of striatal DA tone.^182^, ^183^, ^184^ Importantly, DA is a major regulator of the BG motor circuit (Figure 5), and degeneration of the SNc is correlated with changes in neuronal activity in BG circuits.^186^, ^187^, ^188^, ^189^ Thus, the mainstay of PD treatment over the last five decades has focused on levodopa as a DA replacement agent to normalize the proposed underlying BG circuit dysfunction.^190^

Although effective, long‐term levodopa therapy can eventually lose efficacy or lead to dyskinesia and hallucinations^191^, ^192^ and contribute to impulsivity,^193^ especially when combined with other dopaminergic agents.^194^ For PD with tremor or treatment‐refractory dyskinesias, DBS is an effective therapy for minimizing motor fluctuations,^195^, ^196^, ^197^ where it can be placed in one of the three locations depending on indication and surgical preference. As in ET, DBS of the thalamic VIM is commonly used for tremor suppression in PD. However, VIM‐DBS does not address other symptoms of PD, and DBS of the GPi and STN is frequently used with added benefit for bradykinesia and rigidity.^195^, ^198^, ^199^, ^200^, ^201^ Indeed, the hypothesis that targeting BG circuit dysfunction may improve motor symptoms in patients with PD is supported by the use of GPi‐ and STN‐DBS, with recent studies demonstrating reduction in PD‐associated action and rest tremor with both GPi‐ and STN‐DBS, although the former may require more time than STN‐DBS before observation of maximum tremor suppression.^38^, ^199^, ^200^, ^201^ In patients with advanced disease undergoing BG recordings during DBS placement, hyperactivity and increased bursting are consistently observed in the STN and GPi.^202^, ^203^, ^204^ This is in line with observations in nonhuman primates treated with 1,2,3,6‐tetrahydropyridine (MPTP), which produces a DA‐deficient state and parkinsonian‐like symptoms.^27^, ^205^ Moreover, lesioning or DBS of the STN reduces hypokinetic symptoms in MPTP‐treated nonhuman primates.^20^, ^206^ Despite DBS being effective in many patients, the high risk of associated perioperative complications^39^ provides a strong impetus for developing novel agents capable of pharmacologically recapitulating the effects of DBS in PD.

While much of our understanding of the dysfunctional circuitry in PD to date has come from observations of abnormal bursting activity in primate models or in patients with PD, data comparing the brain activity of patients with PD to healthy participants, or to patients with PD before disease onset, are lacking. In one study comparing advanced and early PD, patients with more advanced PD were shown to have neurons that fire at a higher rate while demonstrating higher overall level of STN background activity.^207^ In the absence of neuronal recordings from healthy brain, studies across different disease populations can provide added insight. A study comparing STN activity in patients with PD versus ET (a disease without known involvement of the BG) showed that the mean firing rate in STN neurons was significantly increased in PD compared to ET, with a larger percentage of neurons in PD exhibiting the burst firing phenotype.^208^ Additionally, in a comparison between patients with PD and patients with dystonia (a disease also known to involve the BG), mean GPi discharge rate was also higher in patients with PD.^204^ Thus, despite the lack of available control data, the combined preclinical and clinical evidence to date support the hypothesis of BG circuit hyperactivity in the pathophysiology of PD.

A specific role for TTCCs in modulating the underlying circuit dysfunction in PD can be inferred from preclinical studies, which allude to TTCC involvement in burst firing within BG circuit nodes, notably the STN. The abnormal bursting activity in the BG circuit associated with PD has been causally linked to TTCC activity in preclinical models. A TTCC blocker, ML218, inhibits TTCC current and consequently reduces low threshold spike and rebound burst activity in STN neurons in ex vivo thalamic slices, and further reverses haloperidol‐induced catalepsy in a rat model of PD.^53^ However, ML218 has demonstrated no effect on parkinsonian symptoms in MPTP‐treated monkeys;^52^, ^209^ in part, due to its sedative effects, which highlights the need for well‐tolerated agents. Further evidence for TTCC involvement in parkinsonian symptoms is seen in the 6‐hydroxydopamine (6‐OHDA) unilateral lesion model, which results in unilateral depletion of striatal DA innervation. In rats and mice, such DA depletion leads to STN neuronal hyperactivity and rebound bursts thought to be mediated by TTCCs.^29^, ^210^, ^211^, ^212^ To this end, local infusion of TTCC blockers into the STN of 6‐OHDA lesioned rats, but not L‐type calcium channel blockers, has demonstrated reduced STN bursting activity and improved motor performance.^29^ Another study found that STN‐targeted DBS has differential effects on open field locomotor activity (distance moved, rearing, and movement duration) in 6‐OHDA and healthy rats, whereby delivery of constant negative current reduced STN burst activity and eliminated parkinsonian symptoms in 6‐OHDA rats, while delivery of constant positive current increased STN burst activity and induced a parkinsonian state in healthy rats.^54^ Altogether, these studies suggest a strong relationship between STN burst firing and parkinsonian symptoms that can be modulated by both TTCC blockade and DBS.

While the electrical underpinnings of bradykinesia and rigidity seem to relate, at least in part, to disrupted TTCC modulation of BG motor circuits, the mechanisms underlying PD tremor are less clear. Preclinical PD tremor research is particularly scarce due to the lack of a robust rest tremor model.^213^ Nonetheless, some evidence suggests that aberrant motor circuitry is similarly implicated in tremor genesis in PD. For example, oscillatory activity in the BG has been observed in patients with PD with limb tremor; specifically, neurons with high‐frequency oscillatory activity have been found in the STN and denoted “tremor cells” due to their spike rate correlating with limb tremor frequency.^214^ Notably, recordings derived through thalamic DBS exploration in patients with PD have also identified neuronal subpopulations within the ventral nuclear thalamus,^215^ VIM, and ventralis oralis posterior in which neuronal activity correlates with tremor as assessed via electromyography sensors.^216^ These findings allude to a potential alternative mechanism for PD tremor implicating TTCC‐rich nodes within the CTC circuit. Indeed, overlapping CTC involvement and oscillatory flow between the cerebellum and sensorimotor cortex have been reported in patients with PD and ET.^217^ Furthermore, longitudinal functional magnetic resonance imaging in patients with PD has shown increased CTC activity with disease progression, namely greater recruitment of cortical motor‐associated and cerebellar areas.^218^ In an attempt to reconcile a BG versus CTC hypothesis in PD tremor, a “dimmer‐switch” model^219^ has been proposed, which postulates that the BG circuit initiates the tremor episodes, whereas the CTC circuit propagates and produces the tremor. This hypothesis is consistent with findings from a study comparing the functional connectivity of the BG (GPi, external globus pallidus [GPe], putamen, and caudate) and CTC circuits in patients with PD with tremor and those without;^220^ wherein the GPi, GPe, and putamen were activated at the start of tremor episodes, while CTC activity was aligned with tremor amplitude.

As with ET, few studies have focused on the effectiveness of TTCC blockers in PD tremor. In the rat tacrine‐induced tremulous jaw movement model (used to mimic parkinsonian tremor),^221^ mibefradil, a non‐CNS‐penetrant TTCC blocker, has demonstrated largely no effect on tremor suppression, while NNC 55‐0396, a CNS‐penetrant TTCC blocker analog, has been shown to reduce tremor.^222^ In MPTP monkeys with levodopa‐responsive tremor, ethosuximide, an anticonvulsant with TTCC blocking properties,^111^, ^223^ has demonstrated tremor suppression.^224^ Together, these studies lend support to the hypothesis that motor deficits in PD, including both hypokinetic and tremor symptoms, may be reduced via central TTCC blockade. Ongoing and future clinical efforts investigating the safety and efficacy of novel TTCC blockers in patients with PD will be critical to better establish the plausibility of these mechanistic relationships.

## Conclusions and Future Directions

**Figure 6:**
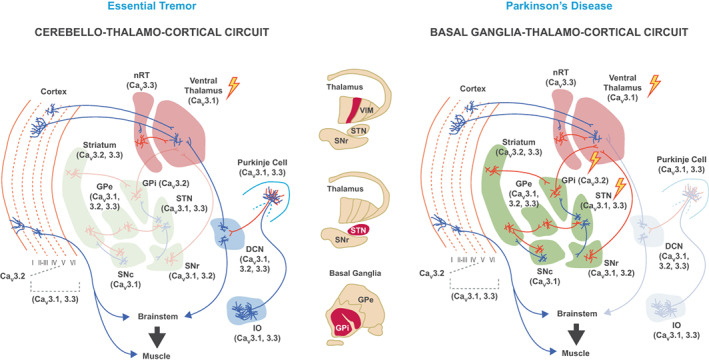
Targets for pharmacologic intervention in ET and PD. (*Middle*, *top to bottom*) The VIM has a high concentration of TTCCs and is a common target for DBS placement in patients with ET. In patients with PD, targets for DBS placement include the VIM, STN, or GPi, all areas with high expression of TTCCs. VIM‐DBS is commonly used for tremor suppression in PD, while the GPi and STN are the preferred targets for managing tremor‐dominant PD, with added benefit for bradykinesia and rigidity. Pharmacologic TTCC blockers therefore have the potential to mimic the effects of DBS as an alternative to surgical intervention in ET and PD. (*Left*) TTCC isoforms (Ca_V_3.1, Ca_V_3.2, and Ca_V_3.3) expressed in olivocerebellar (blue shading) and thalamocortical (pink shading) nodes, with key CTC circuit targets for potential pharmacologic intervention in ET highlighted. Inhibitory and excitatory neurons are denoted by red and blue lines, respectively. (*Right*) TTCC isoforms (Ca_V_3.1, Ca_V_3.2, and Ca_V_3.3) expressed in BG (green shading) and thalamocortical nodes (pink shading), with key BG circuit targets for potential pharmacologic intervention in PD highlighted. Inhibitory and excitatory neurons are denoted by red and blue lines. DCN, deep cerebellar nuclei; GPe, external globus pallidus; GPi, internal globus pallidus; nRT, nucleus reticularis of the thalamus; IO, inferior olive; SNc, substantia nigra compacta; SNr, substantia nigra reticulata; STN, subthalamic nucleus. *Adapted from Park et al*.^86^ *as licensed under CC BY 4.0*.

The concept of treating ET and PD as diseases of dysfunctional circuitry represents an important paradigm shift in the management of movement disorders. The development of agents with novel mechanisms of action that can pharmacologically mimic the effects of DBS has the potential to provide much needed alternatives to invasive surgical interventions. While identifying suitable targets for pharmacologic intervention has been challenging, the current mechanistic exploration of ET and PD points to consistent patterns of disrupted circuitry, including cerebellar and thalamic rhythmic oscillations in ET and STN burst firing in PD. Importantly, TTCC activity appears to be implicated in both ET and PD circuit pathophysiology, with TTCC blockade emerging as a promising therapeutic strategy for alleviating the motor symptoms of these movement disorders (Figure 6). Multiple trials have been completed or are now underway to investigate the safety and efficacy of TTCC blockers in ET and PD. Suvecaltamide (JZP385/CX‐8998, NCT03101241),^176^ PRAX‐944 (NCT05021978),^177^ and NBI‐827104 (NCT04880616) have been assessed in patients with moderate to severe ET. Additional Phase 2 trials in ET are currently underway for suvecaltamide (NCT05122650) and PRAX‐944 (NCT05021991).^178^ PRAX‐944 is also being evaluated in patients with PD with motor fluctuations. Future investigations including those aimed at improved selectivity for certain Ca_V_ isoforms may lead to the development of treatments targeted to critical neuronal circuit nodes in ET and PD.

We conclude with some considerations for future investigational efforts. To successfully measure the effect of TTCC blockers on motor symptoms, studies need to be designed carefully to (i) optimize CNS target engagement and drug titration, and (ii) monitor/minimize potential adverse events. Firstly, differences in efficacy among TTCC blockers may reflect differences in CNS penetrance, with some agents more able to cross the blood–brain barrier. Assessing target engagement in response to TTCC blockade via CNS pharmacodynamic biomarkers^55^ may therefore be useful for informing dose range finding in clinical trials. Secondly, TTCCs have been linked to hormone secretion, neurotransmitter release, sleep–wake cycles, and feeding behavior, with many other physiologic roles outside the CNS, including vascular and cardiomyocyte functions.^89^, ^225^, ^226^, ^227^, ^228^, ^229^ Thus, attenuation of aberrant neural activity with minimal disruption to normal TTCC activity should be a therapeutic objective when developing novel agents for ET and PD. Relatedly, drug‐target affinity determination will be an important consideration for future drug development efforts. Taken together, these recommendations provide a critical framework for the development of much needed agents capable of targeting the dysfunctional circuitry underlying pathologic conditions such as ET, PD, and beyond.

## Author Contributions

Conceptualization and literature review: Lillian G. Matthews, Corey B. Puryear, Susana S. Correia, Sharan Srinivasan, Gabriel M. Belfort, Ming‐Kai Pan, and Sheng‐Han Kuo. Analysis and interpretation: Lillian G. Matthews, Corey B. Puryear, Susana S. Correia, Sharan Srinivasan, Gabriel M. Belfort, Ming‐Kai Pan, and Sheng‐Han Kuo. Visualization: Lillian G. Matthews, Corey B. Puryear, Sharan Srinivasan, Gabriel M. Belfort, and Sheng‐Han Kuo. Writing—original draft: Lillian G. Matthews, Corey B. Puryear, Susana S. Correia, and Sharan Srinivasan. Writing—review and editing: Lillian G. Matthews, Corey B. Puryear, Susana S. Correia, Sharan Srinivasan, Gabriel M. Belfort, Ming‐Kai Pan, and Sheng‐Han Kuo. All authors read and approved the final article.

## Funding information

Funding for editorial support was provided by Praxis Precision Medicines. SHK is supported by NINDS #R01 NS104423, NINDS #R01 NS118179, and NINDS #R01 NS124854.

## Conflict of Interest Statement

LGM serves as a paid consultant to Praxis Precision Medicines and is a Praxis shareholder. CBP, GMB, and SC were employees of Praxis Precision Medicines during article preparation and are Praxis shareholders. MKP and SHK serve as scientific advisors for Praxis Precision Medicines and Sage Therapeutics.

## Supporting information


Figure S1
