# Supplementary material for: T‐type calcium channels as therapeutic targets in essential tremor and Parkinson's disease
Source: Ann Clin Transl Neurol. 2023 Feb 4;10(4):462–83. doi: 10.1002/acn3.51735 (PMC10109288; doi:10.1002/acn3.51735)
Supplement: Supplementary file 1 — Figure S1 [file ACN3-10-462-s001.docx]

**
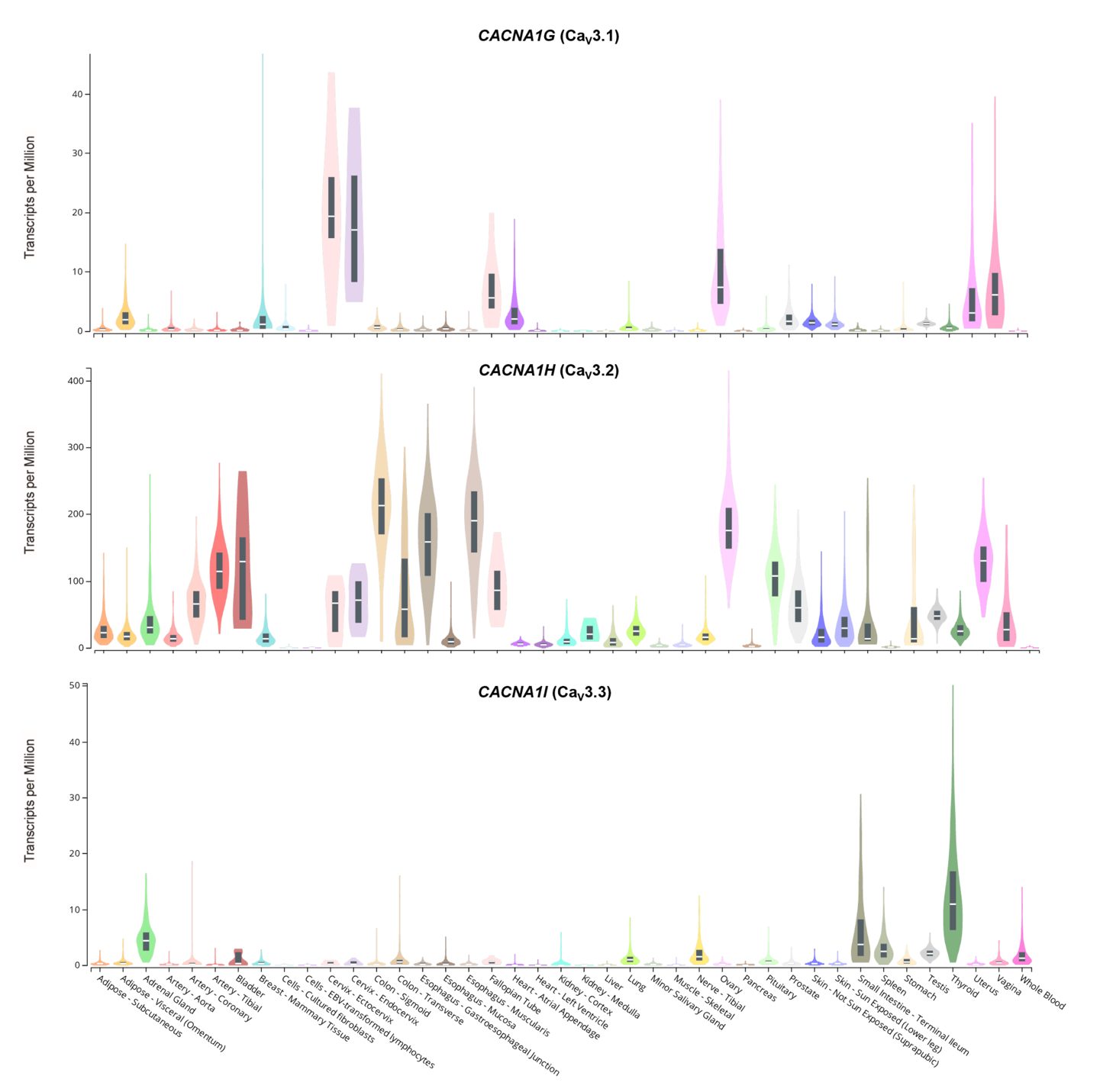
**

**Supplementary Figure 1.** Violin plots depicting *in situ* hybridization-based gene expression of *CACNA1G* (Ca_V_3.1), *CACNA1H* (Ca_V_3.2), and *CACNA1I* (Ca_V_3.3) in human peripheral tissue. Gene expression values are shown in transcripts per million, calculated from a gene model with isoforms collapsed to a single gene. No other normalization steps have been applied. Box plots are shown as median and 25^th^ and 75^th^ percentiles. *Plots generated and adapted from The Genotype-Tissue Expression (GTEx) Project portal.*
